# Recognizing IgG4-related tubulointerstitial nephritis

**DOI:** 10.1186/s40697-016-0126-5

**Published:** 2016-07-17

**Authors:** Shawna Mann, Michael A. Seidman, Sean J. Barbour, Adeera Levin, Mollie Carruthers, Luke Y. C. Chen

**Affiliations:** Department of Medicine, University of British Columbia, 2775 Laurel Street, 10th Floor, Vancouver,, BC V5Z 1M9 Canada; Department of Pathology and Laboratory Medicine, Providence Health Care, 1081 Burrard Street, Vancouver, BC V6Z 1Y6 Canada; Division of Nephrology, University of British Columbia, 2775 Laurel Street, 5th Floor, Vancouver, BC BC V5T 3A5 Canada; Division of Nephrology, University of British Columbia, 1081 Burrard Street, Vancouver, BC V6Z 1Y6 Canada; Division of Rheumatology, University of British Columbia, 839 West Broadway, Vancouver, BC V5Z 1J9 Canada; Division of Hematology, University of British Columbia, 2775 Laurel Street, 10th Floor, Vancouver, BC V5Z 1M9 Canada

**Keywords:** IgG4-related disease, IgG4-related kidney disease, Tubulointerstitial nephritis, IgG4-related tubulointerstitial nephritis, Autoimmune tubulointerstitial nephritis, Epidemiology

## Abstract

**Purpose of the review:**

Immunoglobulin G4-related disease (IgG4-RD) is a systemic fibroinflammatory disorder affecting nearly all organs, including the kidney. Tubulointerstitial nephritis (IgG4-TIN) is the most common form of IgG4-related kidney disease (IgG4-RKD) and is the focus of this concise review.

**Objective:**

The study aims to describe when IgG4-TIN should be suspected and to summarize the diagnosis, treatment, and natural history of the disease.

**Sources of information:**

Ovid MEDLINE, Google Scholar, and PubMed were searched for full-text English language articles up to January 2016. References included in the manuscript were chosen at the authors’ discretion based on their relevance to the subject of the review.

**Findings:**

IgG4-TIN should be considered in patients presenting with abnormal urinalysis, abnormal kidney function, renal lesions on imaging, and elevated IgG, IgE, or hypocomplementemia. Diagnosis of IgG4-TIN requires a combination of histologic features (plasma cell-enriched TIN with >10 IgG4+ plasma cells/hpf, +/− TBM immune complex deposits in many cases) and at least one of the following:

- Characteristic radiologic findings (small peripheral low-attenuation cortical nodules, round or wedge-shaped lesions, or diffuse patchy involvement)
- Elevated serum IgG4 level
- Characteristic findings of IgG4-RD in other organs

Other conditions such as lupus, vasculitis, diabetic nephropathy, and lymphoma must be excluded, as these can also present with IgG4+ plasma cells in the renal parenchyma. IgG4-TIN is generally responsive to steroids and B cell depletion with rituximab, but relapses are common and patients require close long-term follow-up.

**Limitations:**

Available data on IgG4-TIN are from retrospective observational studies.

**Implications:**

IgG4-TIN is a distinct and emerging subtype of interstitial nephritis. Nephrologists must be aware of this entity and how to definitively diagnose and treat it. Prospective studies and ideally multi-center clinical trials are needed to study the epidemiology, treatment, and natural history of this disease.

## Résumé

**Motif de la revue:**

Les maladies associées aux immuglobulines G4 (IgG4) sont caractérisées par un désordre systémique d’ordre fibro-inflammatoire qui affecte pratiquement tous les organes, incluant les reins. La néphropathie tubulo-interstitielle (*tubulointerstitial nephritis: IgG4-TIN*), qui fait l’objet de la présente revue, est la maladie rénale associée aux IgG4 la plus fréquemment observée.

**Objectif de la revue:**

On a voulu faire le portrait de la néphropathie tubulo-interstitielle (IgG4-TIN) telle que nous la comprenons actuellement: les signes cliniques indiquant sa possible présence, les indices permettant son diagnostic, ainsi que le traitement et l’évolution naturelle de cette maladie.

**Sources:**

Tous les articles publiés en anglais sur ce sujet jusqu’en janvier 2016 ont été répertoriés sur Ovid MEDLINE, Google Scholar et PubMed. Les références choisies pour la présente revue ont été sélectionnées à la discrétion des auteurs, sur la base de leur pertinence au sujet.

**Constat:**

Une IgG4-TIN devrait être suspectée chez les patients présentant des anormalités lors d’une analyse d’urine, des troubles de la fonction rénale, des lésions aux reins observables par imagerie, des taux élevés d’IgG, d’IgE ou une hypocomplémentémie. Le diagnostic d’une IgG4-TIN demande la combinaison de paramètres histologiques (infiltrat tubulo-interstitiel riche en plasmocytes, avec >10 plasmocytes IgG4+ par HPF, avec ou sans dépôts de complexes immuns dans la membrane basale tubulaire) ainsi qu’au moins une des manifestations suivantes:

- l’observation radiologique d'indices caractéristiques de la maladie (nodules corticaux périphériques de faible atténuation, cunéiformes ou d’atteinte inégale et diffuse)
- des taux élevés d’IgG4 sériques
- la présence d’une maladie associée aux IgG4 dans d’autres organes

Les maladies telles que le lupus, la vascularite, la néphropathie diabétique ou les lymphomes sont exclues puisque celles-ci sont également caractérisées par une présence élevée de plasmocytes sécréteurs d’IgG4 au niveau du parenchyme rénal. De façon générale, l’IgG4-TIN répond bien aux stéroïdes et à une déplétion des lymphocytes B avec le rituximab, mais les récidives sont fréquentes et les patients requièrent un suivi étroit et à long terme.

**Limites de l’étude:**

Les données colligées sur la néphropathie tubulo-interstitielle pour cette revue proviennent d’études observationnelles rétrospectives.

**Conclusion:**

L’IgG4-TIN est un sous-type émergent et distinct de néphropathie interstitielle que les néphrologues doivent pouvoir identifier, diagnostiquer et traiter. Des études prospectives, idéalement des essais cliniques impliquant plusieurs centres de soins, sont nécessaires pour étudier l’épidémiologie et déterminer le traitement et l’évolution naturelle de cette maladie.

## Introduction

### Why is this review important?

Immunoglobulin G4-related disease (IgG4-RD) is an emerging disease, which is difficult to diagnose, and remains under-recognized, particularly in patients with single-organ system involvement including IgG4-related kidney disease (IgG4-RKD). Tubulointerstitial IgG-RKD is the most common form of renal involvement and represents an important evolution in the understanding of tubulointerstitial nephritis. While many sub-classifications of glomerulonephritis exist, there are relatively few distinct subtypes of tubulointerstitial nephritis.

### What are the key messages?

1. Tubulointerstitial nephritis (IgG4-TIN) is an important emerging subtype of interstitial nephritis.
2. Little is known about the epidemiology of this disease and what proportion of all TIN is represented by IgG4-TIN.
3. Diagnosis requires that clinicians and pathologists recognize the clinical, radiologic, and histologic features and correlate findings with established diagnostic criteria.

### Implications for future research/policy

Research is needed in the epidemiology, treatment, and natural history of tubulointerstitial IgG4-RKD.

## Main text

### Overview of IgG4-related disease

IgG4-RD is a fibroinflammatory disease that can affect almost any organ, with nearly identical histopathologic findings in all tissues: a lymphoplasmacytic infiltrate of polyclonal lymphocytes, IgG4+ plasma cells, and eosinophils [1, 2], often most prominent in the early stages of disease, and fibrosis, more commonly seen in the chronic phase of the disease. The pathophysiology is complex and incompletely understood; aberrant T helper type 2 (Th2)/T regulatory cells are responsible for profibrotic cytokine production leading to collagen deposition and fibrosis [3–5]. The most common clinical manifestations are autoimmune pancreatitis (AIP), sialadenitis, dacryoadenitis, and retroperitoneal fibrosis; other common clinical manifestations are listed in Table 1. Intrinsic renal involvement is found in about 12 % of patients [6].

**Table 1:** Common manifestations of IgG4-RD by organ system

Gastrointestinal	Autoimmune pancreatitis Sclerosing cholangitis Sclerosing mesenteritis
Head and neck	Eosinophilic angiocentric fibrosis (puffy, fibroinflammatory lesions of orbits and upper respiratory tract) Orbital pseudotumor Riedel’s thyroiditis Mikulicz’s disease (enlargement of lacrimal, salivary, and parotid glands) Kuttner’s tumor (salivary gland enlargement)
Allergy/respiratory	Asthma, atopy, allergy Tracheal stenosis Chronic sinusitis Pleural and pulmonary nodules, interstitial lung disease Fibrosing mediastinitis
Systemic	Multifocal fibrosclerosis (orbits, thyroid, retroperitoneum, mediastinum)
Large vessels	Inflammatory aortic aneurysm Periaoritis and periartertitis
Renal/retroperitoneum	Retroperitoneal fibrosis (RPF) Intrinsic renal disease (IgG4-related kidney disease): – Tubulointerstitial nephritis (IgG4-TIN); often with hypocomplementemia – Membranous glomerulonephropathy (IgG4-MGN); anti-PLA2R negative Renal pyelitis
Nervous system	Hypertrophic pachymeningitis Hypophysitis Peri-neural masses
Blood and bone marrow	Eosinophilia Polyclonal hypergammaglobulinemia with elevation in IgG4 and other immunoglobulins

IgG4-RD in general affects middle-aged to elderly patients with a slight male preponderance [3, 7] and can mimic other systemic conditions such as sarcoidosis, lymphoma, and Sjögren’s syndrome. However, the epidemiology may vary depending on the pattern of organ involvement. For example, pulmonary IgG4-related disease affects middle-aged men and women more than the elderly [8, 9], whereas biliary disease is more common in older males [10, 11], and Mikulicz’s disease (IgG4-RD of the lacrimal and salivary glands) tends to affect younger females [12]. IgG4-RD affecting a single-organ system may be initially misdiagnosed as an autoimmune disorder such as nephritis, pancreatitis, or cholangitis.

Definitive diagnosis of IgG4-RD requires an adequate tissue biopsy demonstrating storiform fibrosis, mild to moderate tissue eosinophils, lymphoplasmacytic infiltrate, increased IgG4 levels (generally >50–100/hpf for most tissues), and an IgG4/IgG+ plasma cell ratio >40 % [1, 2, 13]. Measurement of serum IgG4 is important, but is neither sensitive nor specific by itself. Extremely elevated serum IgG4 levels >10–15 g/L are rarely seen outside of IgG4-RD, but moderately elevated serum IgG4 can be seen in a number of inflammatory, autoimmune, and neoplastic conditions; conversely, 30–45 % of patients with histologically confirmed IgG4-RD (usually in a single system) have normal serum IgG4 levels [6, 14]. Clinicians must therefore be sufficiently aware of the clinical, radiologic, and laboratory features to seek tissue biopsy and alert the pathologist when IgG4-RD is suspected. Once diagnosed, IgG4-RD can be treated favorably with steroids and/or rituximab, and the highly variable degrees of recovery appear dependent on the extent of fibrosis at the time of diagnosis [7]. The disease was initially thought to be monophasic when first reported, but cumulative literature now shows that it is typically a chronic disease with frequent relapse. Furthermore, regression of the disease in one organ may be followed by emergence of the disease in another organ. This underscores the need for early diagnosis to limit the extent of irreversible fibrosis and improve patient outcomes.

Despite rising awareness, the epidemiology remains poorly understood and the true incidence and prevalence are likely underestimated [2, 7, 15]. Japanese population-based studies have reported that the prevalence of AIP seems to have risen from 2.2 per 100,000 population in 2007 to 4.6 per 100,000 in 2011 [16, 17]. Most of these patients (87.6 % in 2007 and 86.4 % in 2011) were considered to have IgG4-related (type I) AIP [16, 17]. Whether this rise in prevalence from 2007 to 2011 reflects increased recognition as opposed to increased incidence of new cases is not clear. To our knowledge, there are no similar population-based studies in North American or European populations on any subtype of IgG4-RD, including IgG4-RKD.

Many retrospective studies have revealed that IgG4-RD is responsible for many cases of “idiopathic” or unexplained disease in diverse organs. For example, one review of 26 patients with autoimmune hepatitis revealed that nine had characteristics suggestive of IgG4-RD [18]. Subsequently, the hepatology community has increased efforts to identify IgG4-associated cholangitis, given that the treatment is vastly different from non-IgG4 cholangitis—steroids versus surgery [18–20]. IgG4-RD involvement in many organs, including the kidneys, remains under-recognized.

### Overview of IgG4-related kidney disease

IgG4-RD can affect structures adjacent to the kidney, leading to acute or chronic kidney disease. The most common of such manifestation is retroperitoneal fibrosis leading to obstructive uropathy. Involvement of the renal pelvis in the form of IgG4 pyelitis has been reported but is rare. Intrinsic renal disease is encompassed by the term IgG4-related kidney disease (IgG4-RKD) [21, 22]. In a large case series of 125 patients with IgG4-RD, 23 (18.4 %) had RPF and 15 (12 %) had intrinsic IgG4-RKD [6]. The kidney is unique among organ systems in that there are two histologic subtypes of IgG4-RD involvement: most commonly, tubulointerstitial nephritis (IgG4-TIN) and rarely, membranous glomerulonephropathy (IgG4-MGN). IgG4-MGN is reported in about 10 % of cases of IgG-RKD and may occur with or without TIN. Unlike idiopathic membranous glomerulonephropathy, IgG4-MGN is not associated with anti-PLA2R antibodies [23].

Patients with IgG4-RKD are demographically similar to those with IgG4-RD in general, most commonly middle aged to elderly with a male preponderance [7, 24]. Some patients present with isolated renal disease, but involvement of other organs, most commonly sialadenitis and lymphadenopathy, is common. In a large American series, 28 of 34 patients (83 %) with IgG4-TIN had another organ affected by IgG4-RD at the time of diagnosis by Kawano and Saeki and Raissian et al. [24, 25], and in a Japanese series, extra-renal lesions were present in 42 of 43 patients (98 %) [26].

### IgG4-TIN: clinical presentation and radiologic and histologic findings

The clinical presentation of tubulointerstitial IgG4-RKD is variable, and renal function may range from normal to severely compromised [21, 26]. Out of 35 patients with IgG4-TIN from the Mayo Clinic study, 27 (77 %) had acute or progressive chronic renal failure at the time of their renal biopsies [25]. Unlike medication-related TIN, IgG4-TIN may not be associated with an increase in urinary WBCs or WBC casts [24]. IgG4-TIN causes local tissue inflammation, but systemic acute phase reactants tend to be normal or minimally elevated; thus, unlike other forms of TIN, markedly elevated CRP is rarely observed [24]. Most patients (70–88 %) with TIN related to IgG4-RKD have elevated serum IgG and/or IgG4 levels [24, 25]. At the Mayo Clinic, 56 % of patients with IgG4-TIN presented with hypocomplementemia [25] compared to 20 % of patients with IgG4-RD without renal involvement. On contrast CT scan, typical lesions are small, bilateral, nodular, or wedge-shaped, in the renal parenchyma diffusely or in the peripheral cortex [27, 28]. The lesions are low-enhancing on both contrast CT and T2-weighted MRI [27]. Notably, they are isointense on T1-weighted MRI and are not visible on non-contrast CT scan [27]. Less commonly, they may be extra-parenchymal (in the renal sinus, pelvis, or surrounding the renal cortex), or take on the appearance of masses or cysts [27, 29]. MRI with diffusion-weighted imaging (DWI) will enhance the ability to diagnose IgG4-RKD, as the hyperintense DWI lesions appear to be detected with 100 % sensitivity as compared to the lower sensitivity (77.4 %) of T2-weighted MRI [28]. Mass lesions or enlarged kidneys may be seen initially and atrophic kidneys may be seen later in the disease course.

**Fig. 1:**
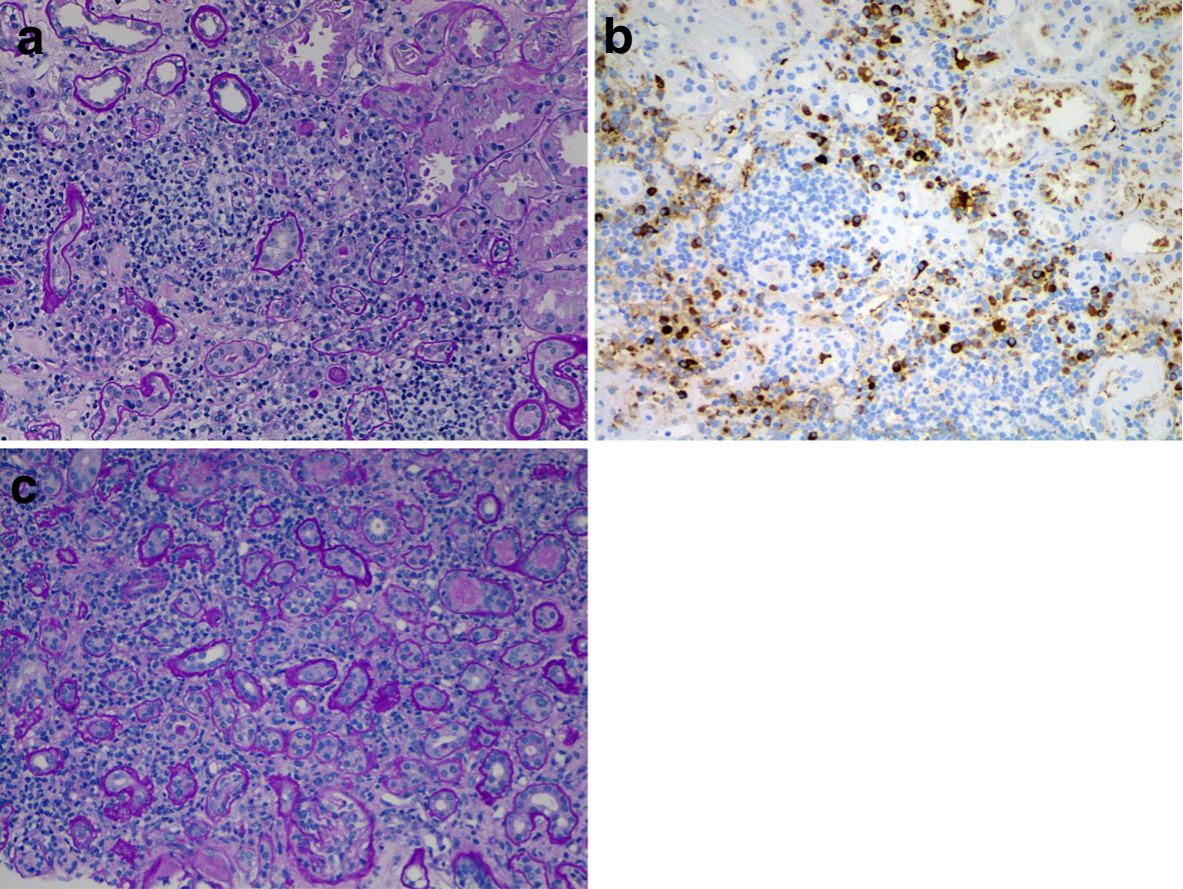
IgG4-related tubulointerstitial nephritis (IgG4-TIN). **a** Periodic acid-Schiff (PAS) stain of a case of clinically confirmed IgG4-related kidney disease (IgG4-RKD) showing tubulointerstitial nephritis with a significant number of plasma cells and a plasma cell tubulitis. **b** IgG4 immunohistochemistry confirming a large number of IgG4 expressing plasma cells in the infiltrate. **c** For comparison, periodic acid-Schiff (PAS) stain of a case of obstructive uropathy due to benign prostatic hypertrophy (BPH), showing a similarly cellular interstitial infiltrate but with fewer plasma cells

**Fig. 2:**
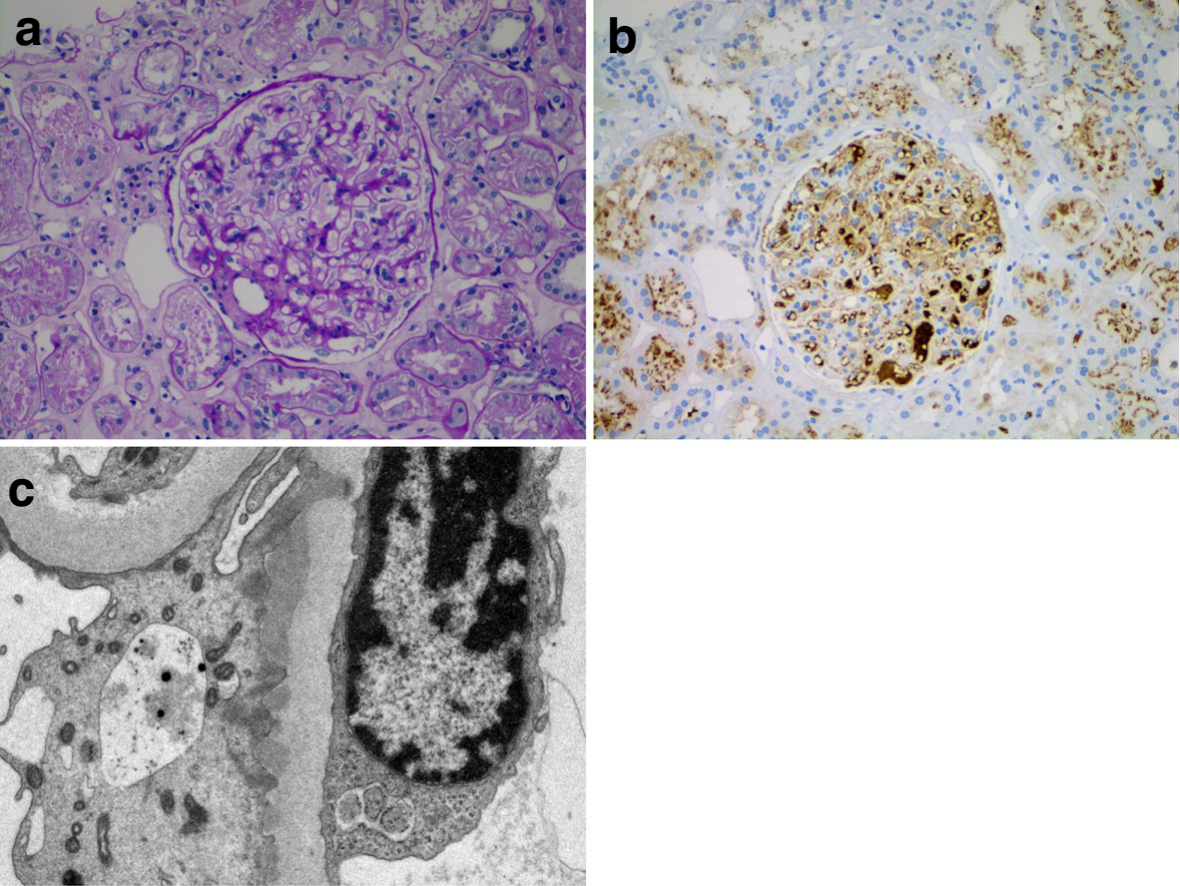
IgG4-related membranous glomerulonephropathy. **a** Periodic acid-Schiff (PAS) stain of a case of clinically confirmed IgG4-related kidney disease (IgG4-RKD, same patient as in Fig. 1), showing a membranous pattern of glomerulonephropathy with occasional plasma cells in the mesangium. **b** IgG4 immunohistochemistry confirming several IgG4 expressing plasma cells in the glomerulus. **c** Electron microscopy demonstrating subepithelial electron dense deposits, consistent with membranous glomerulonephropathy

Histology is of primary importance to diagnosing IgG4-RKD. Of the many organs affected by IgG4-RD, the kidney is unique in having two distinct patterns of histologic involvement: TIN (Fig. 1) and, less commonly, membranous glomerulonephropathy (Fig. 2). The following diagnostic features have been described in both American and Japanese populations: diffuse or multifocal TIN, with variability in the ratio of fibrosis to inflammation; moderate to marked interstitial increase in IgG4+ plasma cells; and increased mononuclear cells with eosinophils in most, but not all, cases [13, 21, 25, 30, 31]. Mononuclear cell tubulitis, if present, is focal and mild, and plasma cell tubulitis is rarely seen. Glomeruli are normal or have mild mesangial matrix expansion, and there may be tubular basement membrane immune complex deposits, made up of polyclonal IgG and complement C3. Simply looking for plasma cell infiltration would lead to over-diagnosis, and only looking at biopsies with moderate to severe interstitial inflammation may lead to under-diagnosis [25]. Ruling out lupus, vasculitis, lymphoma, and other causes of kidney conditions is crucial, as increased IgG4+ plasma cells have been reported in the renal parenchyma in various diseases [32]. A Mayo Clinic study reviewed 414 biopsies taken over a 1-year period which had moderate to severe inflammation [25]. Of those, 20 % were found to have increased plasma cells (defined as >5 plasma cells/×400 magnification hpf in ≥3 fields). Of those cases, the authors found that 2 % were subsequently confirmed to represent IgG4-TIN, 76 % were ascribed to other etiologies, and 22 % remained unable to classify. Their data suggest that while IgG4-TIN is a rare cause of moderate to severe interstitial inflammation with accompanying plasma cells, the prevalence in patients with mild interstitial inflammation and plasma cell infiltrates is unknown. The Mayo Clinic has proposed a diagnostic framework for IgG4-TIN, outlined in Table 2 [25]. These criteria should be considered complementary to the International Consensus Criteria for IgG4-RD [13], which are the standard diagnostic criteria for IgG4-RD in general.

**Table 2:** Mayo Clinic proposed diagnostic criteria of IgG4-TIN

TIN IgG4-RKD can be confirmed by the presence of tubulointerstitial nephritis with >10 IgG4+ plasma cells/hpf in the most concentrated field, plus at least one of the following:	
• Histology:	
ο Tubular basement membrane immune complex deposits by immunofluorescence, immunohistochemistry, and/or electron microscopy	
• Imaging:	
ο Small peripheral low-attenuation cortical nodules, round or wedge-shaped lesions, or diffuse patchy involvement	
• Serology:	
ο Elevated serum IgG4 or total IgG level	
• Other organ involvement such as the following:	
ο AIP, sclerosing cholangitis, inflammatory masses of any origin, sialadenitis, inflammatory aortic aneurysm, lung involvement, retroperitoneal fibrosis	

IgG4-TIN should be considered in patients with AIN who do not have an obvious pharmacologic or infectious etiology, and it should be considered in patients with systemic symptoms potentially consistent with extra-renal IgG4-RD, and when there are findings of other organ involvement characteristic of IgG4-RD. Renal imaging showing low-attenuating lesions in the kidneys should also prompt suspicion of IgG4-TIN. When IgG4-TIN is suspected, the renal biopsy should be examined for a lymphoplasmacytic infiltrate, and if increased plasma cells are present, these should be stained for IgG and IgG4. These stains can also be performed on archived tissue samples from kidney and other organs, as many of these patients have chronic disease with previous “non-diagnostic” biopsies. Given the non-specific nature of the histologic appearance of IgG4-TIN, the pathologist should be specifically informed of a high degree of clinical suspicion for IgG4-RD in order to trigger appropriate immunohistochemical evaluation. Serum IgG, IgG subclasses, serum protein electrophoresis, complement levels, and imaging for multiple and bilateral low-attenuating renal lesions should also be considered.

When a renal biopsy demonstrates TIN and increased IgG4+ plasma cells, other diagnoses to consider include pauci-immune glomerulonephritis, medication-induced interstitial nephritis, autoimmune TIN, lupus, Sjögren’s syndrome, and chronic pyelonephritis. Once pauci-immune GN is ruled out, the IgG4 stain has a sensitivity of 100 % and specificity of 92 % for IgG4-RKD [25]. Other features detailed in Table 2 can assist in ruling in or out IgG4-TIN. Early steroid responsiveness is characteristic of IgG4-TIN, but this feature is of limited diagnostic value given that several other diseases on the differential diagnosis are also steroid responsive.

### IgG4-TIN: treatment and natural history

The treatment of IgG4-TIN mirrors the treatment of IgG4-RD in general. The 2015 International Consensus Statement for treatment of IgG4-RD advocates treating active disease urgently and that many patients with asymptomatic disease also require treatment to prevent or minimize future organ damage [33]. Glucocorticoids for IgG4-RD are first line, and a typical regimen is prednisone 0.6–1 mg/day × 4 weeks with a slow taper thereafter and maintenance prednisone in many cases [33]. A prospective single-arm Japanese study of 57 patients reported an overall response rate of 82 % with a relapse rate of 12 % with such a regimen [34]. The main toxicity was new or worsening diabetes in 30 % of patients. The general strategy of first-line steroid treatment has been retrospectively examined in Japanese patients with IgG4-TIN, and most patients have a clinically significant degree of sustained renal recovery [26, 35, 36]. While steroids are the standard first-line therapy in Japan, B cell depletion with rituximab is also very potent. A prospective, single-arm clinical trial examining the effect of two doses of rituximab (1 g IV rituximab 2 weeks apart) demonstrated a clinical response in 29 of 30 patients [37]. Of these, 22 patients had a sustained disease response at 12 months while 7 relapsed during that period, requiring re-treatment with rituximab. Steroid-sparing agents such as azathioprine, mycophenolate mofetil, 6-mercaptopurine, methotrexate, tacrolimus, or cyclophosphamide have been used, mainly in the setting of AIP, with limited success [38]. Rituximab is effective in most cases refractory to steroids and other immunomodulators, although rare cases requiring more intensive therapy such as purine analogues have been reported [39]. A recent case report confirmed the effectiveness of rituximab in a steroid-refractory patient with IgG4-TIN, with sustained improvement in renal function [40]. Two other case reports suggest that treatment of steroid-refractory renal disease may be successful when low-dose prednisolone is combined with either and azathioprine or mizoribine [41].

With respect to prognosis, most patients’ renal function improves once treatment is initiated, and this tends to plateau after weeks to months or may slightly decline from its peak recovery [35, 42]. As in other organs affected by IgG4-related fibrosis, fibrosis is generally not reversible. Some patients may still have fibrotic lesions in the tubulointerstitium after treatment despite improvements in renal function, imaging, and other histologic findings [26, 27, 36, 40]. Serum IgG4, hypocomplementemia, and proteinuria are helpful disease markers to follow for relapse, especially if present initially [26]. Quantitative flow cytometry of peripheral blood plasmablasts has shown promise as a more sensitive disease marker than serum IgG4, but this test is not routinely available in most centers [43]. Radiographic improvement is variable: in a Japanese review, 18 of 40 patients had radiographic recovery [26], whereas in a Mayo study, 10 of 13 patients experienced radiographic resolution of their renal lesions [27]. In general, patients with a lower estimated glomerular filtration rate (eGFR) at time of diagnosis (<60 mL/min) tend to progress more frequently than those whose eGFR is >60 mL/min, despite treatment [26, 44], but even in those with significant renal dysfunction (eGFR <60 mL/min), a 1-month course of steroids resulted in significant improvement [36]. While some patients go on to develop dialysis dependence [36], the number of patients with end-stage renal disease as a result of IgG4-TIN is unknown. There is a paucity of data on renal transplant, but anecdotally, one patient in our center was transplanted for end-stage renal disease due to IgG4-MGN in November 2014 and remains in remission on prednisone 2.5 mg daily as well as mycophenolate mofetil and tacrolimus with monthly monitoring of kidney function, serum IgG, and IgG4 (unpublished data).

In summary, many patients will respond to steroids, with new or worsening diabetes as the most significant toxicity, and rituximab is a potent and well-tolerated treatment option both for steroid naïve and steroid-refractory patients. Other traditional steroid-sparing immunosuppressives have been used with variable success, and their role is not clearly defined. Many patients will require maintenance prednisone or re-treatment with rituximab, and our own practice is either to maintain steroid-responsive patients on a low dose of prednisone or for those patients who achieve a steroid-free complete response with rituximab, to monitor serum IgG, IgG4, and kidney function closely and plan for re-treatment at the earliest sign of relapse.

## Conclusions

Interstitial nephritis is a frequent finding in patients biopsied for acute or chronic kidney disease [45, 46]. Causes of interstitial nephritis such as medications, autoimmune disease, and infections are likely far more common than IgG4-TIN. However, many TIN cases currently labeled “autoimmune” or “idiopathic” may represent unrecognized IgG4-TIN, and improved detection of IgG4-TIN in these patients is a priority. With better recognition and prospectively collected data, increased understanding of the epidemiology, natural history, and optimal treatment of patients with IgG4-TIN is expected.

## Abbreviations

IgG4-RD, immunoglobulin G4-related disease; IgG4-RKD, immunoglobulin G4-related kidney disease; IgG4-TIN, immunoglobulin G4 tubulointerstitial nephritis; TIN, tubulointerstitial nephritis

## References

[1] Chen L (2015). Does this patient have IgG4 related disease?. Journal of Canadian Rheumatology Association.

[2] Mahajan VS, Mattoo H, Deshpande V, Pillai SS, Stone JH (2014). IgG4-related disease. Annu Rev Pathol.

[3] Umehara H, Nakajima A, Nakamura T, Kawanami T, Tanaka M, Dong L, Kawano M (2014). IgG4-related disease and its pathogenesis-cross-talk between innate and acquired immunity. Int Immunol.

[4] Della-Torre E, Lanzillotta M, Doglioni C (2015). Immunology of IgG4-related disease. Clin Exp Immunol.

[5] Zaidan M, Cervera-Pierot P, de Seigneux S, Dahan K, Fabiani B, Callard P, Ronco P, Aucouturier P (2011). Evidence of follicular T-cell implication in a case of IgG4-related systemic disease with interstitial nephritis. Nephrol Dial Transplant.

[6] Wallace ZS, Deshpande V, Mattoo H, Mahajan VS, Kulikova M, Pillai S, Stone JH (2015). IgG4-related disease: clinical and laboratory features in one hundred twenty-five patients. Arthritis Rheumatol.

[7] Stone JH, Zen Y, Deshpande V (2012). IgG4-related disease. N Engl J Med.

[8] Inoue D, Zen Y, Abo H, Gabata T, Demachi H, Kobayashi T, Yoshikawa J, Miyayama S, Yasui M, Nakanuma Y, Matsui O (2009). Immunoglobulin G4-related lung disease: CT findings with pathologic correlations. Radiology.

[9] Matsui S, Hebisawa A, Sakai F, Yamamoto H, Terasaki Y, Kurihara Y, Waseda Y, Kawamura T, Miyashita T, Inoue H, Hata N, Masubuchi H, Sugino K, Kishi J, Kobayashi H, Usui Y, Komazaki Y, Kawabata Y, Ogura T (2013). Immunoglobulin G4-related lung disease: clinicoradiological and pathological features. Respirology.

[10] Hubers LM, de Buy Wenniger LJ M, Doorenspleet ME, Klarenbeek PL, Verheij J, Rauws EA, van Gulik TM, Oude Elferink RP, van de Graaf SF, de Vries N, Beuers U (2015). IgG4-associated cholangitis: a comprehensive review. Clin Rev Allergy Immunol.

[11] Al-Dhahab H, McNabb-Baltar J, Al-Busafi S, Barkun AN (2013). Immunoglobulin G4-related pancreatic and biliary diseases. Can J Gastroenterol.

[12] Zen Y, Nakanuma Y (2010). IgG4-related disease: a cross-sectional study of 114 cases. Am J Surg Pathol.

[13] Deshpande V, Zen Y, Chan JK, Yi EE, Sato Y, Yoshino T, Klöppel G, Heathcote JG, Khosroshahi A, Ferry JA, Aalberse RC, Bloch DB, Brugge WR, Bateman AC, Carruthers MN, Chari ST, Cheuk W, Cornell LD, Fernandez-Del Castillo C, Forcione DG, Hamilos DL, Kamisawa T, Kasashima S, Kawa S, Kawano M, Lauwers GY, Masaki Y, Nakanuma Y, Notohara K, Okazaki K, Ryu JK, Saeki T, Sahani DV, Smyrk TC, Stone JR, Takahira M, Webster GJ, Yamamoto M, Zamboni G, Umehara H, Stone JH (2012). Consensus statement on the pathology of IgG4-related disease. Mod Pathol.

[14] Carruthers MN, Khosroshahi A, Augustin T, Deshpande V, Stone JH (2015). The diagnostic utility of serum IgG4 concentrations in IgG4-related disease. Ann Rheum Dis.

[15] Beyer G, Schwaiger T, Lerch MM, Mayerle J (2014). IgG4-related disease: a new kid on the block or an old acquaintance?. United European Gastroenterol J.

[16] Kanno A, Masamune A, Okazaki K, Kamisawa T, Kawa S, Nishimori I, Tsuji I, Shimosegawa T (2015). Nationwide epidemiological survey of autoimmune pancreatitis in Japan in 2011. Pancreas.

[17] Kanno A, Nishimori I, Masamune A, Kikuta K, Hirota M, Kuriyama S, Tsuji I, Shimosegawa T (2012). Nationwide epidemiological survey of autoimmune pancreatitis in Japan. Pancreas.

[18] Chung H, Watanabe T, Kudo M, Maenishi O, Wakatsuki Y, Chiba T (2010). Identification and characterization of IgG4-associated autoimmune hepatitis. Liver Int.

[19] Joshi D, Webster G (2014). IgG4 related sclerosing cholangitis. Advances in Hepatology.

[20] Umehara H, Okazaki K, Masaki Y, Kawano M, Yamamoto M, Saeki T, Matsui S, Sumida T, Mimori T, Tanaka Y, Tsubota K, Yoshino T, Kawa S, Suzuki R, Takegami T, Tomosugi N, Kurose N, Ishigaki Y, Azumi A, Kojima M, Nakamura S, Inoue D (2012). A novel clinical entity, IgG4-related disease (IgG4RD): general concept and details. Mod Rheumatol Jpn Rheum Assoc.

[21] Saeki T, Kawano M (2014). IgG4-related kidney disease. Kidney Int.

[22] Cortazar FB, Stone JH (2015). IgG4-related disease and the kidney. Nat Rev Nephrol.

[23] Alexander MP, Larsen CP, Gibson IW, Nasr SH, Sethi S, Fidler ME, Raissian Y, Takahashi N, Chari S, Smyrk TC, Cornell LD (2013). Membranous glomerulonephritis is a manifestation of IgG4-related disease. Kidney Int.

[24] Kawano M, Saeki T (2015). IgG4-related kidney disease—an update. Clin Nephrol.

[25] Raissian Y, Nasr SH, Larsen CP, Colvin RB, Smyrk TC, Takahashi N, Bhalodia A, Sohani AR, Zhang L, Chari S, Sethi S, Fidler ME, Cornell LD (2011). Diagnosis of IgG4-related tubulointerstitial nephritis. J Am Soc Nephrol.

[26] Saeki T, Kawano M, Mizushima I, Yamamoto M, Wada Y, Nakashima H, Homma N, Tsubata Y, Takahashi H, Ito T, Yamazaki H, Saito T, Narita I (2013). The clinical course of patients with IgG4-related kidney disease. Kidney Int.

[27] Takahashi N, Kawashima A, Fletcher JG, Chari ST (2007). Renal involvement in patients with autoimmune pancreatitis: CT and MR imaging findings. Radiology.

[28] Kim B, Kim JH, Byun JH, Kim HJ, Lee SS, Kim SY, Lee MG (2014). IgG4-related kidney disease: MRI findings with emphasis on the usefulness of diffusion-weighted imaging. Eur J Radiol.

[29] Fukuhara H, Taniguchi Y, Matsumoto M, Kuroda N, Fukata S, Inoue K, Fujimoto S, Terada Y, Shuin T (2014). IgG4-related tubulointerstitial nephritis accompanied with cystic formation. BMC Urol.

[30] Saeki T, Nishi S, Imai N, Ito T, Yamazaki H, Kawano M, Yamamoto M, Takahashi H, Matsui S, Nakada S, Origuchi T, Hirabayashi A, Homma N, Tsubata Y, Takata T, Wada Y, Saito A, Fukase S, Ishioka K, Miyazaki K, Masaki Y, Umehara H, Sugai S, Narita I (2010). Clinicopathological characteristics of patients with IgG4-related tubulointerstitial nephritis. Kidney Int.

[31] Kawano M, Saeki T, Nakashima H, Nishi S, Yamaguchi Y, Hisano S, Yamanaka N, Inoue D, Yamamoto M, Takahashi H, Nomura H, Taguchi T, Umehara H, Makino H, Saito T (2011). Proposal for diagnostic criteria for IgG4-related kidney disease. Clin Exp Nephrol.

[32] Houghton DC, Troxell ML (2011). An abundance of IgG4+ plasma cells is not specific for IgG4-related tubulointerstitial nephritis. Mod Pathol.

[33] Khosroshahi A, Wallace ZS, Crowe JL, Akamizu T, Azumi A, Carruthers MN, Chari ST, Della-Torre E, Frulloni L, Goto H, Hart PA, Kamisawa T, Kawa S, Kawano M, Kim MH, Kodama Y, Kubota K, Lerch MM, Lohr M, Masaki Y, Matsui S, Mimori T, Nakamura S, Nakazawa T, Ohara H, Okazaki K, Ryu JH, Saeki T, Schleinitz N, Shimatsu A, Shimosegawa T, Takahashi H, Takahira M, Tanaka A, Topazian M, Umehara H, Webster GJ, Witzig TE, Yamamoto M, Zhang W, Chiba T, Stone JH (2015). International Consensus Guidance Statement on the management and treatment of IgG4-related disease. Arthritis Rheumatol.

[34] Masaki Y, Shimizu H, Sato Nakamura T, Nakamura T, Nakajima A, Iwao Kawanami H, Miki M, Sakai T, Kawanami T, Fujita Y, Tanaka M, Fukushima T (2014). IgG4-related disease: diagnostic methods and therapeutic strategies in Japan. J Clin Exp Hematop.

[35] Saeki T, Kawano M, Mizushima I, Yamamoto M, Wada Y, Ubara Y, Nakashima H, Ito T, Yamazaki H, Narita I, Saito T (2016). Recovery of renal function after glucocorticoid therapy for IgG4-related kidney disease with renal dysfunction. Clin Exp Nephrol.

[36] Mizushima I, Yamada K, Fujii H, Inoue D, Umehara H, Yamagishi M, Yamaguchi Y, Nagata M, Matsumura M, Kawano M (2012). Clinical and histological changes associated with corticosteroid therapy in IgG4-related tubulointerstitial nephritis. Mod Rheumatol.

[37] Carruthers MN, Topazian MD, Khosroshahi A, Witzig TE, Wallace ZS, Hart PA, Deshpande V, Smyrk TC, Chari S, Stone JH (2015). Rituximab for IgG4-related disease: a prospective, open-label trial. Ann Rheum Dis.

[38] Hart PA, Kamisawa T, Brugge WR, Chung JB, Culver EL, Czako L, Frulloni L, Go VL, Gress TM, Kim MH, Kawa S, Lee KT, Lerch MM, Liao WC, Lohr M, Okazaki K, Ryu JK, Schleinitz N, Shimizu K, Shimosegawa T, Soetikno R, Webster G, Yadav D, Zen Y, Chari ST (2013). Long-term outcomes of autoimmune pancreatitis: a multicentre, international analysis. Gut.

[39] Chen LY, Wong PC, Noda S, Collins DR, Sreenivasan GM, Coupland RC (2015). Polyclonal hyperviscosity syndrome in IgG4-related disease and associated conditions. Clin Case Rep.

[40] McMahon BA, Novick T, Scheel PJ, Bagnasco S, Atta MG (2015). Rituximab for the treatment of IgG4-related tubulointerstitial nephritis: case report and review of the literature. Medicine.

[41] Miyata KN, Kihira H, Haneda M, Nishio Y (2014). IgG4-related tubulointerstitial nephritis associated with membranous nephropathy in two patients: remission after administering a combination of steroid and mizoribine. Case Rep Nephrol.

[42] Tsubata Y, Akiyama F, Oya T, Ajiro J, Saeki T, Nishi S, Narita I (2010). IgG4-related chronic tubulointerstitial nephritis without autoimmune pancreatitis and the time course of renal function. Intern Med.

[43] Wallace ZS, Mattoo H, Carruthers M, Mahajan VS, Della Torre E, Lee H, Kulikova M, Deshpande V, Pillai S, Stone JH (2015). Plasmablasts as a biomarker for IgG4-related disease, independent of serum IgG4 concentrations. Ann Rheum Dis.

[44] Kawano M, Saeki T (2015). IgG4-related kidney disease—an update. Curr Opin Nephrol Hypertens.

[45] Muriithi AK, Leung N, Valeri AM, Cornell LD, Sethi S, Fidler ME, Nasr SH (2015). Clinical characteristics, causes and outcomes of acute interstitial nephritis in the elderly. Kidney Int.

[46] Praga M, Sevillano A, Aunon P, Gonzalez E (2015). Changes in the aetiology, clinical presentation and management of acute interstitial nephritis, an increasingly common cause of acute kidney injury. Nephrol Dial Transplant.

